# Editorial: AI in digital oncology: imaging and wearable technology for cancer detection and management

**DOI:** 10.3389/frai.2025.1662971

**Published:** 2025-07-29

**Authors:** Souptik Barua, Channing J. Paller, Navkiran Randhawa, Arvind Rao

**Affiliations:** ^1^Division of Precision Medicine, Department of Medicine, NYU Grossman School of Medicine, New York, NY, United States; ^2^Department of Medical Oncology, Johns Hopkins University School of Medicine, Baltimore, MD, United States; ^3^Department of Medicine, Medical College of Georgia, Augusta University, Augusta, GA, United States; ^4^Department of Computational Medicine and Bioinformatics, University of Michigan Medical School, Ann Arbor, MI, United States; ^5^Department of Radiation Oncology, University of Michigan Medical School, Ann Arbor, MI, United States; ^6^Department of Biostatistics, University of Michigan Medical School, Ann Arbor, MI, United States

**Keywords:** digital health, artificial intelligence (AI), cancer, deep learning, machine learning, histopathology, AI literacy

## Introduction

Cancer remains the second leading cause of death in the United States, with persistent disparities affecting minority groups and individuals with limited healthcare access. While mortality rates have declined for many cancer types, others like pancreatic and thyroid cancers continue to show stagnant or increasing rates. This Research Topic explores how digital health tools, artificial intelligence (AI), machine learning (ML), and computational techniques can address challenges in early cancer detection, diagnosis, treatment planning, and monitoring—with particular focus on reducing healthcare disparities through technological advancement.

## Key contributions and clinical innovations

The papers in this Research Topic demonstrate remarkable progress in applying digital health and AI to cancer care, offering novel solutions to longstanding clinical challenges.

### Addressing digital health disparities

Pana et al.'s study of 270 digital health technology (DHT) users in Romania revealed that DHT users had 2–6 times higher cancer screening rates than the general population, with uptake concentrated among internet-literate subgroups. This highlights both the promise and peril of digital health innovations—while empowering proactive disease management, they risk widening gaps for digitally marginalized populations. The authors advocate systematic integration of DHT tools into primary care workflows to bridge educational and logistical barriers.

### AI applications in gastrointestinal cancers

Akbari et al. provide a comprehensive review of AI applications across esophageal, gastric, hepatocellular, colorectal, and pancreatic cancers. Their analysis reveals ML models achieved on average 89% accuracy in analyzing endoscopic and CT images, with convolutional neural networks demonstrating particular strength in identifying polyps during colonoscopy. However, they emphasize that successful clinical integration requires addressing data quality issues, interpretability concerns, and standardization needs through multidisciplinary collaboration.

### Precision immunotherapy using AI

Wang et al. developed a Transformer-Unet deep learning network to predict PD-L1 expression from routine H&E-stained lung cancer images, eliminating the need for costly immunohistochemical staining. Their model achieved 80% dice similarity coefficient and 0.92 correlation with gold standard assessment—exceeding consistency between different pathologists. This breakthrough could democratize access to precision immunotherapy by making biomarker assessment available in resource-limited settings.

### AI performance in hepatocellular carcinoma (HCC)

Al-Obeidat et al.'s meta-analysis of seven studies comparing AI models with physicians in HCC detection found comparable sensitivity (93%) but highlighted complementary strengths. Region-based convolutional neural networks showed particularly high sensitivity (96%), while physicians maintained superior specificity (100%). This reinforces AI's role as an effective “second reader” rather than replacement for human expertise.

### Computational tumor microenvironment analysis

Khanduri et al. used multiplex immunofluorescence and spatial analytics to map macrophage subtypes in colorectal cancer liver metastases. They discovered that M2 macrophages predominated at tumor periphery with greater proximity to malignant cells, while higher M1 macrophage densities in tumor centers paradoxically correlated with poorer survival—challenging traditional immunology paradigms and demonstrating how computational approaches can reveal prognostic biomarkers invisible to conventional assessment.

## The next frontier: generative AI and multi-agent systems

The evolution toward generative AI and multi-agent systems represents a paradigm shift from isolated AI applications toward comprehensive, coordinated intelligence spanning the entire cancer care ecosystem.

### Generative AI applications

Large language models can synthesize vast medical literature, generate personalized patient explanations, and create adaptive interfaces accommodating varying health literacy levels. For populations with limited digital access—a concern highlighted in Pana et al.'s work—generative AI could provide conversational interfaces requiring minimal technical sophistication while delivering personalized cancer risk information.

### Multi-agent cancer care workflows

Unlike monolithic AI models, multi-agent systems comprise specialized components collaborating through structured workflows. A comprehensive cancer care system might include:

- **Screening agents** analyzing routine imaging and EHR data for elevated cancer risk
- **Diagnostic agents** specialized in different modalities (radiology, pathology, genomics)
- **Treatment planning agents** incorporating molecular profiling and trial evidence
- **Monitoring agents** tracking treatment response and surveillance
- **Patient communication agents** generating personalized educational materials

This distributed architecture offers several advantages: modularity allowing individual agent optimization, transparency through decomposed reasoning steps, robustness through diverse modeling approaches, and adaptive resource allocation for critical tasks.

For example, Wang et al.'s PD-L1 prediction work could be handled by a dedicated histopathology agent while other agents focus on radiologic or genomic aspects, with integration agents synthesizing findings into comprehensive treatment recommendations.

## AI literacy across the care continuum

Successful AI integration requires cultivating AI literacy—understanding AI capabilities, limitations, and appropriate applications—across all stakeholders.

**Healthcare Provider Literacy** encompasses critical evaluation of AI outputs, appropriate patient communication about AI-derived insights, and awareness of potential biases. Training programs must integrate AI literacy into medical education at all levels, emphasizing complementary human-AI collaboration rather than algorithmic deference.

**Patient and Caregiver Literacy** focuses on understanding AI's role in care decisions, appropriate trust calibration, and privacy implications. Educational materials should use accessible language that empowers rather than mystifies, particularly important given digital literacy's influence on healthcare engagement demonstrated in Pana et al.'s findings.

**Health System Literacy** requires governance frameworks for responsible deployment, quality assurance processes, and health equity impact assessments to prevent algorithmic biases from perpetuating cancer care disparities.

## Implementation challenges and future directions

Despite promising results, significant challenges remain in translating AI from research to clinical practice:

### Data quality and standardization

AI performance depends heavily on training data quality and representativeness. Standardizing collection and processing protocols across institutions remains a critical hurdle, particularly for diverse histological image data and physician interpretation variability. Such standardization approaches need contextual consideration and appropriate refinement for subsequent data-derived models and their associated inferences/predictions as well. Concepts like “model-scorecards” (Cherian et al., 2024), data ontologies and conformal inference approaches (Mitchell et al., 2019) might be relevant to bring into our collective knowledge-base in this regard.

### Interpretability and trust

Many AI models function as “black boxes,” challenging clinical acceptance. Multi-agent architectures offer promising solutions by decomposing complex reasoning into transparent, discrete steps performed by specialized components.

### Population diversity

Most AI models are trained on specific demographic groups, raising generalizability concerns. Future work must prioritize diverse data inclusion and explicit performance assessment across demographic groups to ensure equitable benefit rather than exacerbated disparities.

### Workflow integration

Successful implementation requires seamless integration with existing clinical workflows through user-friendly interfaces, clear uncertainty communication, and decision support systems augmenting rather than replacing human expertise.

## Reducing disparities through technology

DHTs and AI technologies offer multiple pathways to extend high-quality cancer care to underserved populations: automating labor-intensive diagnostic tasks (reducing specialized expertise requirements), enabling remote assessment and triage, standardizing care quality regardless of provider experience, and supporting earlier detection across all populations.

However, realizing these benefits requires thoughtful implementation considering access, affordability, and cultural appropriateness—ensuring DHTs and AI reduce rather than widen existing healthcare gaps.

## Conclusion: a vision for equitable AI-enhanced cancer care

This Research Topic illustrates DHT and AI's transformative potential across cancer detection and monitoring pipelines. The emergence of generative AI and multi-agent systems promises evolution from isolated applications toward comprehensive, coordinated intelligence spanning the entire cancer care ecosystem.

Looking forward, we envision seamlessly integrated DHT and AI technologies supporting early detection, accurate diagnosis, personalized treatment planning, and effective monitoring while augmenting rather than replacing human expertise. Multi-agent workflows offer particularly promising approaches for orchestrating complex cancer care pathways with maintained transparency and interpretability.

Critically, this vision includes equitable access to DHT and AI-enhanced cancer care for all populations, regardless of geographic location, socioeconomic status, or demographic characteristics. By thoughtfully developing and implementing these technologies with attention to disparities, we can advance both technical capabilities and meaningful progress toward health equity.

The research presented here represents significant steps toward this vision. Through continued collaboration between computer scientists, oncologists, and healthcare professionals, we can create a future where DHTs and AI help reduce cancer burden for all patients. The frontier of early cancer detection is here—the challenge now is shaping it wisely, inclusively, and with unwavering commitment to improving patient lives while ensuring digital and AI literacy becomes as fundamental to cancer care as the technologies themselves.
